# Lack of knowledge regarding the misuse of domperidone in Pakistan and its serious consequences: short communication

**DOI:** 10.1097/MS9.0000000000000723

**Published:** 2023-04-27

**Authors:** Asifa Kalwar, Rabel Q. Mangi, Junaid U. Rehman, Sidhant Ochani, Muhammad Faraz, Sandhaya Kukreja, Md. Al Hasibuzzaman

**Affiliations:** a Department of Medicine, Dow University of Health Sciences, Karachi; b Department of Medicine, Khairpur Medical College, Khairpur Mir’s; c Department of Medicine, Shaheed Mohtarma Benazir Bhutto Medical University, Larkana, Pakistan; d Institute of Nutrition and Food Science, University of Dhaka, Dhaka, Bangladesh

**Keywords:** domperidone, Pakistan, knowledge, misuse, cardiovascular diseases

## Abstract

Domperidone is an antagonist of the peripheral dopamine (D2) receptor. It works as an antiemetic by blocking D2-receptors at the chemoreceptor trigger zone and as a gastroprokinetic drug by blocking GI tract D2-receptors. According to research, using domperidone significantly raises the risk of cardiac arrhythmia and sudden cardiac death by 70%, most likely through prolonging the QT interval. Blockade of hERG voltage-gated potassium channels is thought to be the reason. Here in Pakistan, this drug is being prescribed by every other physician and even patients frequently self-medicate themselves with it. Due to the serious side effects of this medication, extreme caution should be exercised when prescribing it, especially to the elderly, those who have underlying QT prolongation, those taking medications known to prolong QT, and even more so in pregnant women as there is some evidence that domperidone crosses into breast milk in small amounts and causes an irregular heartbeat in the baby. At least we, on our part, can limit the usage of the drug only with a prescription and, where necessary, if not completely, stop it.

Domperidone is an antagonist of the peripheral dopamine (D2) receptor. It works as an antiemetic by blocking D2-receptors at the chemoreceptor trigger zone and as a gastroprokinetic drug by blocking GI tract D2-receptors. This drug is used in emetogenic chemotherapy-related nausea and vomiting as well as dyspepsia, gastroesophageal reflux, diabetic gastropathy, the production of breast milk, and the prevention of gastrointestinal and emetic side effects of antiparkinsonian medications. Dry mouth, abdominal pain, diarrhea, headache, drowsiness, insomnia, akathisia, extrapyramidal symptoms, and hyperprolactinemia are some of its adverse effects, but its influence on the electrical activity of the heart needs serious attention^1–3^. According to research, using domperidone significantly raises the risk of cardiac arrhythmia and sudden cardiac death by 70%, most likely through prolonging the QT interval^4,5^. Blockade of hERG voltage-gated potassium channels is thought to be the reason^6,7^.

In Pakistan, this drug is being prescribed by every other physician for a lot of conditions causing nausea and vomiting and is often self-medicated because it is readily available over the counter and sold at every pharmacy without the need for any prescription. Apart from low awareness about the adverse effects of domperidone, one of the main contributors to this serious issue in the nation is unauthorized medical professionals operating clinics and pharmacies^8^. This illicit practice of selling drugs in Pakistan is at its zenith, due to which about 0.5 million people die each year in Pakistan^9^. Regarding awareness among physicians about the side effects of domperidone, a survey was conducted for RMM (risk minimization measures) in developed countries like the UK and France, among many others showing minimal awareness (4%) regarding domperidone contraindications^10^. Taking this into consideration, the situation would be even worse in a third-world country like Pakistan lacking a proper healthcare system. Unfortunately, literature is scarce on individual and drug data in Pakistan on domperidone misuse because of the lack of directories. However, as practicing physicians, we have experienced this drug being recommended to patients without taking their proper history to rule out its contraindications, owing to a lack of awareness or negligence on our part. Due to the serious side effects of this medication, extreme caution should be exercised when prescribing it, especially to the elderly, those who have underlying QT prolongation, those taking medications known to prolong QT, and even more so in pregnant women as there is some evidence that domperidone crosses into breast milk in small amounts and causes an irregular heartbeat in the baby^11^.

The Korea Ministry of Food and Drug Safety issued a safety alert in 2014 advising doctors to consider unfavorable cardiac effects when administering domperidone. Following the safety warning, the frequency of domperidone prescriptions in South Korea significantly decreased from 603 962 points in 2011 to 24 623 points in 2016. It was also more safely prescribed to elderly patients regarding the frequency of prescriptions, the maximum daily dose, and the duration of the continuous prescription^12^. The drug regulatory authority of Pakistan decided in 2019 that manufacturers and importers of drugs containing domperidone should update the prescribing information and patient information per the EMA’s recommendation after the European Medicines Agency (EMA) and Agency for the Safety of Medicines and Health Products (ANSM), France, confirmed the serious adverse effects of domperidone^13^.

Unfortunately, the use of domperidone still as candy in Pakistan reflects our healthcare practitioners’ lack of knowledge and no proper checks and balances by the Government in dispensing the drug. Effective policies should be made by the Government to resolve this issue. Unauthorized individuals running clinics and pharmacies should be suspended with immediate effect. Healthcare professionals should be thoroughly educated about the adverse effects of this drug so that they prescribe this medication when necessary only and counsel patients against the frequent self-medication of this drug. Moreover, the Government should use means like newspapers, TV broadcasting, and short text message alerts to create awareness regarding the adverse effects of this drug among the general public. It should be emphasized that domperidone should only be used to manage nausea and vomiting at the lowest possible dose and for the shortest amount of time possible, especially in people who already have cardiac issues. People should be advised to visit their doctor right away if they feel any heart-related issues, such as dizziness, palpitations, chest pain, or an irregular heartbeat. Cardiovascular status assessment before treatment can be pretty important in the current healthcare system. Even better would be to stop prescribing this medication because some developed nations, including Canada, Belgium, and many more, have demanded its removal from the market following compelling research tying it to premature cardiac deaths^14^. At least we, as physicians, on our part, can limit the usage of the drug only with a prescription and, where necessary, if not completely, stop it.

## Ethical approval

Not applicable.

## Consent

Not applicable.

## Sources of funding

The author(s) received no financial support for the research, authorship, and/or publication of this article.

## Author contribution

A.K.: conceptualized the topic; A.K., R.Q.M., and J.U.R.: literature review; A.K., R.Q.M., J.U.R., S.O., M.F., and S.K.: wrote the manuscript; M.F. and S.K.: formatting and referencing; S.O. and M.A.H.: review – editing.

## Conflicts of interest disclosure

The author(s) declared no potential conflicts of interest concerning the research, authorship, and/or publication of this article.

## Provenance and peer review

Not commissioned, externally peer-reviewed.
